# High power laser-driven ceramic phosphor plate for outstanding efficient white light conversion in application of automotive lighting

**DOI:** 10.1038/srep31206

**Published:** 2016-08-09

**Authors:** Young Hyun Song, Eun Kyung Ji, Byung Woo Jeong, Mong Kwon Jung, Eun Young Kim, Dae Ho Yoon

**Affiliations:** 1 School of Advanced Materials Science & Engineering, Sungkyunkwan University, Suwon 440-746, Republic of Korea; 2 SKKU Advanced Institute of Nanotechnology (SAINT), Sungkyunkwan University, Suwon 440-746, Korea; 3 LG Electronics, Material & Device Advanced Research Institute Advanced Optics Team, Seoul 137-724, Korea; 4 Hyosung Corporation, R & D Business Labs, Anyang 431-080, Republic of Korea

## Abstract

We report on Y_3_Al_5_O_12_: Ce^3+^ ceramic phosphor plate (CPP) using nano phosphor for high power laser diode (LD) application for white light in automotive lighting. The prepared CPP shows improved luminous properties as a function of Ce^3+^ concentration. The luminous properties of the Y_3_Al_5_O_12_: Ce^3+^ CPP nano phosphor are improved when compared to the Y_3_Al_5_O_12_: Ce^3+^ CPP with bulk phosphor, and hence, the luminous emittance, luminous flux, and conversion efficiency are improved. The Y_3_Al_5_O_12_: Ce^3+^ CPP with an optimal Ce^3+^ content of 0.5 mol % shows 2733 lm/mm^2^ value under high power blue radiant flux density of 19.1 W/mm^2^. The results indicate that Y_3_Al_5_O_12_: Ce^3+^ CPP using nano phosphor can serve as a potential material for solid-state laser lighting in automotive applications.

Solid state lighting (SSL) has attracted much interest since the first development of light emitting diodes (LED) using blue-emitting InGaN by S. Nakamura in 1995^1^. White LEDs are good candidates as lighting devices capable of replacing incandescent and fluorescent lamps due to their outstanding properties, such as long lifetimes, high luminance and compactness^2^,^3^,^4^,^5^,^6^. Generally, white light can be generated with blue-emitting devices and yellow emitting phosphor (Y_3_Al_5_O_12_: Ce^3+^)^7^. The high efficiency of Y_3_Al_5_O_12_: Ce^3+^ phosphor facilitates the better luminescence performance in white LEDs. However, Y_3_Al_5_O_12_: Ce^3+^ phosphor in white LEDs exhibits problems associated with thermal quenching due to the higher operating current of LEDs^8^. With the decrease of LED efficiency as a function of higher operating current, the temperature of the LEDs increases and this efficiency is lost as heat. The increase of temperature in LEDs has significant effects on the Y_3_Al_5_O_12_: Ce^3+^ phosphor such as a decrease in efficiency and a possible shift of emission wavelength. Overall, the system efficiency of LEDs decreases and it is difficult to apply Y_3_Al_5_O_12_: Ce^3+^ phosphor in high power LEDs. In contrast with LEDs, laser diodes (LD) can easily resolve the issues of LEDs. The output power and external quantum efficiency (EQE) of laser diodes increase linearly as a function of operating current, and the colour stability of the laser emission peak is maintained^9^,^10^. These characteristics make Y_3_Al_5_O_12_: Ce^3+^ phosphor an attractive excitation source for new high-power LD in white light applications. For the generation of white light, the thermal stability of Y_3_Al_5_O_12_: Ce^3+^ phosphor is required in addition to blue LDs. In other words, it is necessary to avoid the use of organic resin with phosphor powders such as epoxy and silicone. An alternative to high power LDs in white light applications is to use the phosphor in glass and ceramic phosphor plates, similar to remote phosphor^11^. Phosphor in glass is not suitable for application to high power LDs because the luminous flux of the phosphor in glass gradually decreases at >1.0 W/mm^2^ due to thermal quenching^12^. Ceramic phosphor plate (CPP) is combined with a laser diode because it is very different from high power blue laser diodes, which provide both optical and thermal stability compared with the mixture of organic resin with phosphor. To fabricate the CPP using bulk-type phosphor, solid state reaction method is required at high temperature^13^. This is a relatively simple method to fabricate the CPP but it is not easy to control the small grain size (<1 μm) due to the initial size of phosphor. To lower the sintering temperature, it is imperative to use the nano-structured Y_3_Al_5_O_12_: Ce^3+^ phosphor. Previous works have demonstrated synthetic techniques such as co-precipitation, sol-gel, and combustion and spray pyrolysis methods^14^,^15^,^16^,^17^. These have been proposed as lower temperature synthesis methods for achieving the nano-structured single phase. Many scientists reported the luminous efficacy of Y_3_Al_5_O_12_: Ce^3+^ CPP in LED applications when using bulk Y_3_Al_5_O_12_: Ce^3+^ 18,19,20. However, the Y_3_Al_5_O_12_: Ce^3+^ CPP for white light generation in blue LD applications such as display, lamp, light fidelity, and automotive lighting sections have yet to be investigated in detail^21^,^22^. In this paper, we report our advanced research on Y_3_Al_5_O_12_: Ce^3+^CPP using nano-structured Y_3_Al_5_O_12_: Ce^3+^ precursor directly combined with blue LDs for white light generation. We substitute the mixture of organic resin with phosphor powder to Y_3_Al_5_O_12_: Ce^3+^ CPP. We also optimize the optical properties of Y_3_Al_5_O_12_: Ce^3+^ CPP.

## Experimental

### Preparation of the Nano-structured Y_3_Al_5_O_12_: Ce^3+^ phosphors

Nano-structured Y_3_Al_5_O_12_: Ce^3+^ phosphors were synthesized using the forced hydrolysis method. First, aluminum nitrate nonahydrate (Sigma-Aldrich, ≥98% pure), aluminum sulfate octadecahydrate (Sigma-Aldrich, ≥98% pure) and urea (Sigma-Aldrich, 99.5%) were used to obtain the nano-structured Al(OH)_3_ particles. These materials were dissolved in deionized water and aged at 98 °C for 4 h. The precipitate was then separated through centrifugation and washed several times with both deionized water and ethanol. Next, yttrium nitrate (Sigma-Aldrich, 99.8%), cerium nitrate (Sigma-Aldrich, 99%), and urea (Sigma-Aldrich, 99.5%) were used to dissolve the above materials in deionized water. The synthesized Al(OH)_3_ particles were homogeneously dispersed in the mixed solution, which was vigorously stirred at 98 °C for 3 h. The precipitate was isolated *via* centrifugation, washed with both deionized water and ethanol, and then dried using a lyophilizer. The resulting powder was annealed at 1200 °C for 4 h under a reducing nitrogen atmosphere containing 5% H_2_ gas. To compare the luminous properties, we synthesized bulk type Y_3_Al_5_O_12_: Ce^3+^ phosphor via solid state reaction method at at 1450 °C for 12 h under same condition.

### Fabrication of the Y_3_Al_5_O_12_: Ce^3+^ CPP

**Figure 1:**
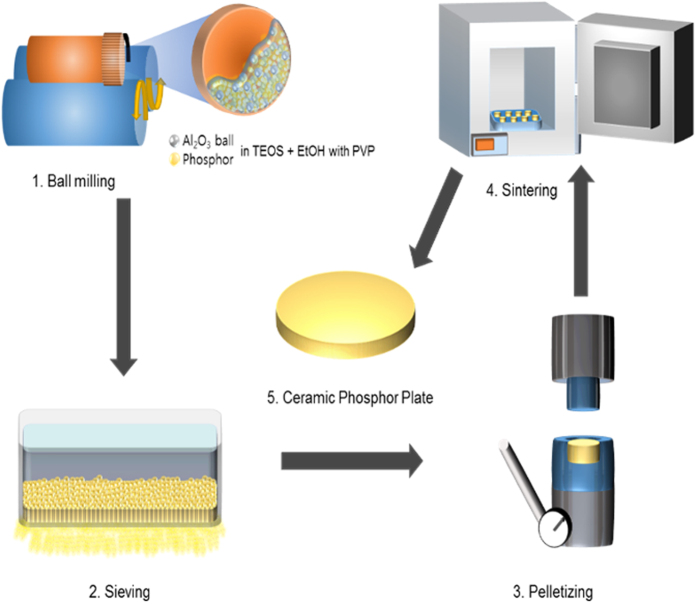
The schematic diagram of process for fabrication of nano-structure based Y_3_Al_5_O_12_: Ce^3+^ ceramic phosphor plate.

The procedure for fabricating the Y_3_Al_5_O_12_: Ce^3+^ CPP is shown in Fig. 1. The fabrication of Y_3_Al_5_O_12_: Ce^3+^CPP was carried out by ball milling nano-structured Y_3_Al_5_O_12_: Ce^3+^phosphor and SiO_2_ as a sintering aid. During this process, ethanol was used as a solvent at a weight ratio of powder:ethanol (1:5). All materials were mixed and milled with a ball milling machine using ZrO_2_ balls with a diameter of 5 mm for 24 h. These mixtures were dried at 90 °C for 12 h and compressed into a pellet using uniaxially pressed under 20 MPa with a diameter of 10 mm and a thickness of 5 mm. The pellets were fired at 1000 °C for 6 h under air atmosphere to remove organic materials and then cold isostatically pressed under 300 MPa for 30 min. The green bodies were sintered with a graphite-heated vacuum furnace (10^−3^ Pa) at 1600 °C for 12 h. The Y_3_Al_5_O_12_: Ce^3+^CPP sintered to remove oxygen vacancies was annealed at 1450 °C for 24 h under air atmosphere. Finally, all samples were mirror-polished on both surfaces.

### Measurements and Characterization

The crystalline phase of the nano-structured Y_3_Al_5_O_12_: Ce^3+^phosphor was identified using powder X-ray diffraction (XRD, D-MAX 2500, Rigaku) with the CuKα target aligned to 10° ≤ 2θ ≤ 80°. The excitation and emission spectra of the nano-structured Y_3_Al_5_O_12_: Ce^3+^ phosphor were analyzed by room-temperature photoluminescence spectrometry (PL, PSI Co., Ltd/Korea), using a 500 W xenon discharge lamp as an excitation source. The surface morphology and the compositions of the Y_3_Al_5_O_12_: Ce^3+^CPP was observed by field-emission scanning electron microscopy (FE-SEM, JSM-7600F, JEOL, Japan). The luminous emittance, luminous flux and conversion efficiency of Y_3_Al_5_O_12_: Ce^3+^CPP was measured with double integrating spheres (PSI Co., Ltd/Korea) under blue laser excitation (3.5 W LD x 8, 445 nm blue LD) using handmade equipment from LG electronics.

## Results and Discussion

**Figure 2:**
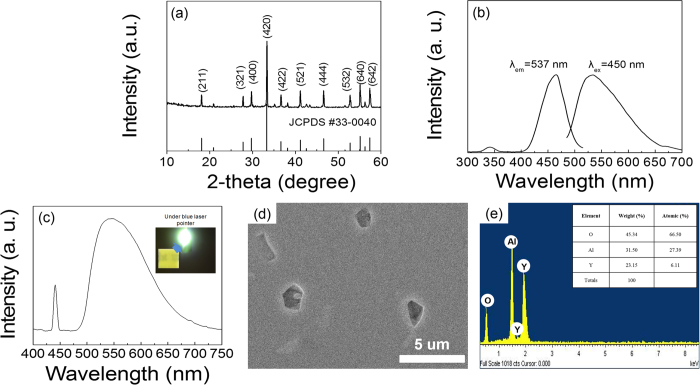
Structure, luminescence, and morphology of fabricated nano-structure based Y_3_Al_5_O_12_: Ce^3+^ ceramic phosphor plate. (**a**) XRD patterns (**b**) PL excitation and emission spectra (**c**) Electroluminescence spectra (**d**) SEM image (**e**) EDX analysis.

The crystal structure of Y_3_Al_5_O_12_: Ce^3+^ CPP was initially analyzed using the powder XRD with a CuKα radiation of λ = 1.5406 Å at 10° ≤ 2*θ* ≤ 80° as shown in Fig. 2a. The XRD patterns of the obtained sample at 2*θ* = 18.1°, 27.6°, 29.7°, 33.3°, 36.4°, 41.0°, 46.6°, 55.1° and 57.4° were completely indexed pure Y_3_Al_5_O_12_ phase (JCPDS No. 33-0040) without any peaks assigned to the CeO_2_, Y_2_O_3_, Al_2_O_3_, YAlO_3_, or Y_2_Al_4_O_9_ phases, which indicate a cubic garnet crystal structure with lattice parameter of 1.2008 nm. The excitation and emission spectra of nano-structured Y_3_Al_5_O_12_: Ce^3+^ phosphor using a xenon lamp as an excitation source are displayed in Fig. 2b. In previous work, the electronic transition of Ce^3+^ ion constitutes 4f^1^ in the ground state and 4f°5d^1^ in the excited state^23^. The ground state is split into a doublet of two excitation bands at 338 and 450 nm, which was attributed to electronic transition from ^2^F_5/2_ to the split excited 5d states band in the ground state of the Ce^3+^ ion^24^. The ground state is split into a doublet of ^2^F_7/2_ and ^2^F_5/2_ because of spin–orbit interactions and the excited state is also split from the crystal field, which is affected by the surrounding ligand ions^25^. Ce^3+^emission involves parity- and spin-allowed 5d → 4f electronic transition and consists of a yellow emitting primary band at 537 nm as well as a shoulder on the longer wavelength side, which is ascribed to electronic transition from 5d → ^2^F_5/2_ and from 5d → ^2^F_7/2_ Ce ions, respectively^26^. Also, in comparison with bulk Y_3_Al_5_O_12_: Ce^3+^ phosphor, the emission wavelength of the nano-structured Y_3_Al_5_O_12_: Ce^3+^ phosphor is shifted to the blue region. This phenomenon is explained as a crystal field strength around Ce^3+^ ion was somewhat reduced^27^. The luminescence in a nano-structured Y_3_Al_5_O_12_: Ce^3+^ phosphor is due to the transition between the energy levels of Ce^3+^ atoms as the luminescent center. After the sintering process, the surface of Y_3_Al_5_O_12_: Ce^3+^CPP (1.5 mm X 1.5 mm) was uniformly mirror-polished with a thickness of 100 μm to convert the highly bright white colour via electroluminescence (EL) spectra as shown in Fig. 2c. Also, the real image of Y_3_Al_5_O_12_: Ce^3+^ CPP under blue laser pointer (1 W) is indicated in the inset in Fig. 2c. The surface imgae of Y_3_Al_5_O_12_: Ce^3+^ CPP is shown in Fig. 2d. EDX composition analyses revealed the presence of oxygen (O), yttrium (Y), and aluminum (Al) elements in Fig. 2e. This result is supported by XRD analysis.

**Figure 3:**
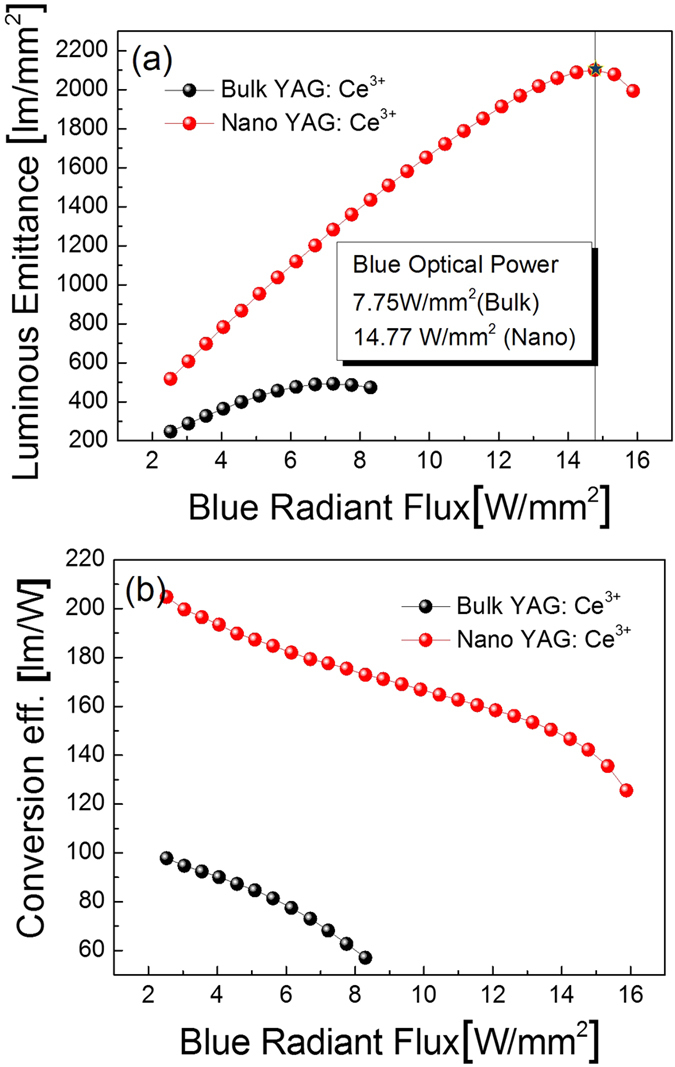
Luminous properties of Y_3_Al_5_O_12_: Ce^3+^ ceramic phosphor plate under a blue laser diode at 445 nm. (**a**) emittance properties (**b**) conversion efficiency, repectively. a.u., arbitrary unit.

The final goal of this study is to use Y_3_Al_5_O_12_: Ce^3+^ CPP in 445 nm emitting blue LDs for white light generation in automotive lighting. To achieve this goal, it is essential to analyze the luminous properties including the luminous emittance, luminous flux, conversion efficiency, emission spectra, CRI, and CCT of the prepared samples under blue laser excitation at 445 nm. Figure 3a presents the luminous emittance of the white LD package under blue laser excitation at 445 nm. With increasing blue incident power, the luminous emittance of Y_3_Al_5_O_12_: Ce^3+^ CPP is linearly increased. This is attributed to the increased number of electrons pumped up to the excited state of Ce^3+^ ions as a function of the increased blue incident power. The maximum luminous emittance value for Y_3_Al_5_O_12_: Ce^3+^ CPP is using the nano-structure Y_3_Al_5_O_12_: Ce^3+^ phosphor obtained at a blue radiant flux density of 14.77 W/mm^2^. This means that the Y_3_Al_5_O_12_: Ce^3+^ ceramic phosphor plate can be applied as a yellow emitting converter in LD applications. However, luminous saturation comes about at high blue radiant flux density due to the thermal quenching effect, which implies that the luminance of the Y_3_Al_5_O_12_: Ce^3+^ CPP cannot be improved further.

Figure 3b depicts the conversion efficiency of Y_3_Al_5_O_12_: Ce^3+^ CPP with increasing blue incident power. When the incident power increases, the conversion efficiency decreases gradually. Both the luminous flux and conversion efficiency of Y_3_Al_5_O_12_: Ce^3+^ CPP using the nano-structure Y_3_Al_5_O_12_: Ce^3+^ phosphor indicates better luminous properties than those of bulk Y_3_Al_5_O_12_: Ce^3+^ phosphor. This is ascribed to the effect of temperature during the fabrication of ceramic phosphor plates. Because the sintering process for ceramic phosphor plates is relatively culminated, the packing density of CPP using nano-structure Y_3_Al_5_O_12_: Ce^3+^ phosphor results in more outstanding luminous properties than for bulk Y_3_Al_5_O_12_: Ce^3+^ in Table 1. In nano-structure Y_3_Al_5_O_12_: Ce^3+^ phosphors, the micro structures of the ceramic phosphor plate are more perfectly formed than bulk Y_3_Al_5_O_12_: Ce^3+^. The densification and grain growth also gradually improved because of higher purity nano precursors, homogeneity, low degree of agglomeration, good crystallinity and low temperature sintering. These results shows that CPP using the nano-structure Y_3_Al_5_O_12_:Ce^3+^ phosphors have a finer and more uniform microstructure, as shown in Supplementary Information Fig. S1.

**Figure 4:**
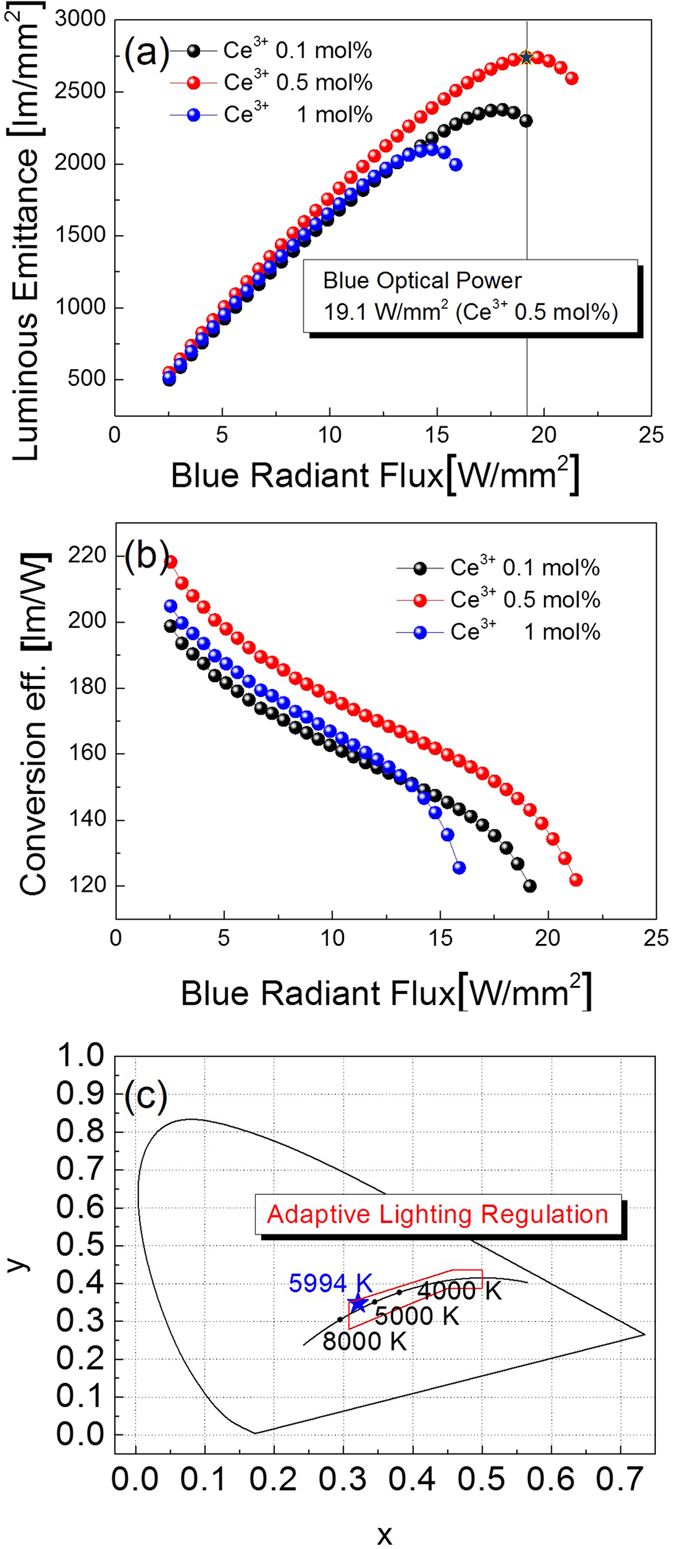
Luminous properties of Y_3_Al_5_O_12_: Ce^3+^ ceramic phosphor plate with increasing the Ce^3+^ ion concentration under a blue laser diode at 445 nm. (**a**) emittance properties (**b**) conversion efficiency, repectively. a.u., arbitrary unit (**c**) CIE colour coordinates.

Figure 4a compares the changes in luminous emittance as a function of Ce^3+^ concentration under a blue laser diode at 445 nm. Luminous emittance increased with increasing amounts of Ce^3+^, up to about 0.5 mol%, and the highest intensity was observed for CPP with 0.5 mol% Ce^3+^ ions. The value of illuminance is 2733 lm/mm^2^. On the other hand, the illuminance value was degraded by 1 mol% Ce^3+^ ions. This means that the luminous emittance decreases with increasing blue incident power at 14.2 W/mm^2^. The Supplementary Information in Fig. S2 suggests that Y_3_Al_5_O_12_: Ce^3+^ can be applied as white conversion materials with high blue incident power density. As demonstrated in Fig. 4b, the white conversion efficiency of Y_3_Al_5_O_12_: Ce^3+^ CPP is improved with increasing blue incident power. When the blue incident power is 19.1 W/mm^2^, the conversion efficiency decreases abruptly from 218 lm/W to 120 lm/W due to thermal quenching. Figure 4c presents the CIE colour coordinates. The calculated CCT value of the nano-structured Y_3_Al_5_O_12_: Ce^3+^ CPP with 0.5 mol% Ce^3+^ ions was 5994 K. This is attributed to the daylight (5500–6000 K) for adaptive lighting regulation.

We believe that CPP using the nano-structure Y_3_Al_5_O_12_:Ce^3+^ will act as a candidate for the automotive lightings.

## Conclusion

In summary, we fabricated Y_3_Al_5_O_12_: Ce^3+^ CPP using nano-structured phosphor under high-power blue radiant flux for white generation in automotive lighting. The synthesis technique, crystal structure, and luminous characteristics are investigated. Nano-structure based Y_3_Al_5_O_12_: Ce^3+^ CPP exhibited an increase in luminous emittance, luminous flux and conversion efficiency compared to Y_3_Al_5_O_12_: Ce^3+^ CPP with bulk phosphor. By applying the Y_3_Al_5_O_12_: Ce^3+^ CPP to blue LD chips, we obtained suitable white light with 2733 lm/mm^2^, 1424.6 lm, 218 lm/W, CRI value of 54.2 and CCT value of 5994 K. We believe that Y_3_Al_5_O_12_: Ce^3+^ CPP using nano phosphor is one of the simplest ways to obtain high luminous properties. Thus, our results suggest that this material can potentially serve as remote phosphors in phosphor converted LDs for white light generation.

## Additional Information

**How to cite this article**: Song, Y. H. *et al.* High power laser-driven ceramic phosphor plate for outstanding efficient white light conversion in application of automotive lighting. *Sci. Rep.* **6**, 31206; doi: 10.1038/srep31206 (2016).

## Supplementary Material

Supplementary Information

## Figures and Tables

**Table 1:** Luminous characteristics of nano-structure based Y_3_Al_5_O_12_: Ce^3+^ ceramic phosphor plate compared with bulk Y_3_Al_5_O_12_: Ce^3+^ one.

Type	CCT	Ra	Chromaticity coordinate		Current (mA)	lm/um
			x	y		
Bulk YAG: Ce^3+^ CPP	9938	53.9	0.289	0.271	400 mA	1.285
Nano-structure YAG: Ce^3+^ CPP	6052	54.2	0.321	0.332	400 mA	2.691

*The rated current of the 445 nm blue laser diode.

Thickness of each phosphor plate: 100 um.
