# Supplementary material for: High power laser-driven ceramic phosphor plate for outstanding efficient white light conversion in application of automotive lighting
Source: Sci Rep. 2016 Aug 9;6:31206. doi: 10.1038/srep31206 (PMC4977504; doi:10.1038/srep31206)
Supplement: Supplementary Information [file srep31206-s1.pdf]

Supporting Information

High power laser-driven ceramic phosphor plate for outstanding efficient white light conversion in application of automotive lighting

Young Hyun Song,[1†] Eun Kyung Ji, [2†] Byung Woo Jeong,[3†] Mong Kwon Jung,[4] Eun Young Kim,[3] and Dae Ho Yoon[1,2,*]

[1]School of Advanced Materials Science & Engineering, Sungkyunkwan University, Suwon 440-746, Republic of Korea.

[2]SKKU Advanced Institute of Nanotechnology (SAINT), Sungkyunkwan University, Suwon 440-746, Korea.

[3]LG Electronics, Material & Device Advanced Research Institute Advanced Optics Team, Seoul 137-724, Korea.

[4]Hyosung Corporation, R&D Business Labs, Anyang 431-080, Republic of Korea.

	Bulk YAG	Nano YAG
structure	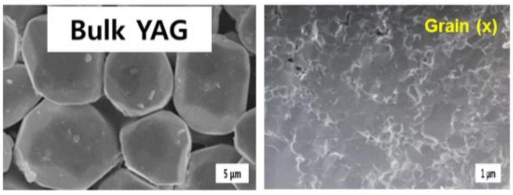	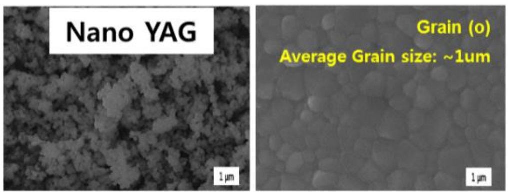

Supplementary Figure 1: The comparison of surface analysis in the prepared CPP.

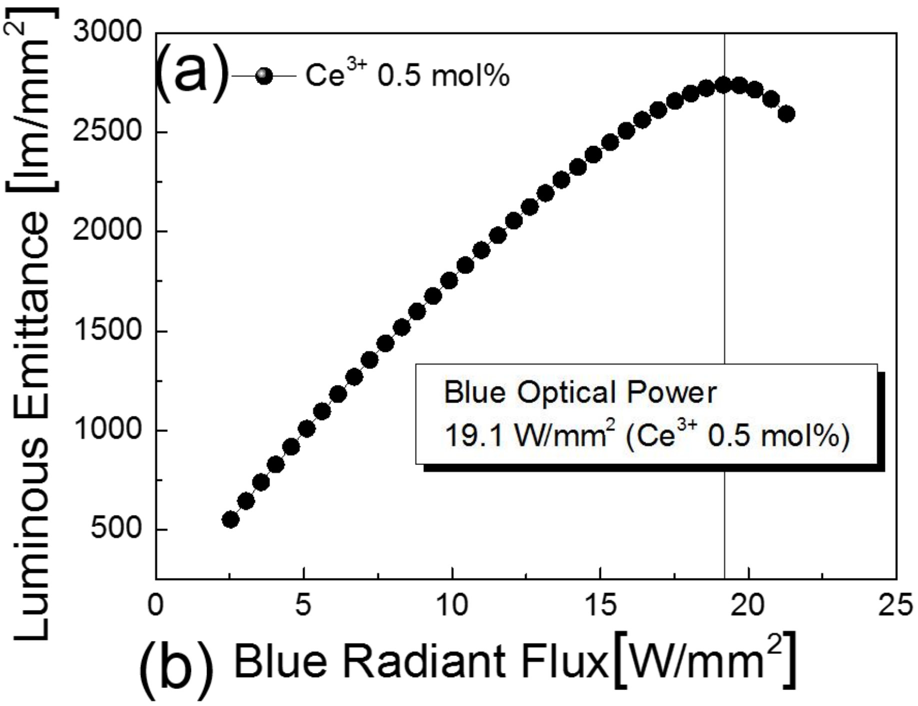

(b)

Type	CCT	Chromaticity coordinate x	y	Light Distribution Image
Ce^{3+} 0.5 mol%	5994	0.321	0.349	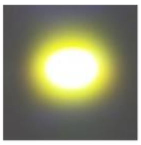

Supplementary Figure 2: luminous properties of Ce^{3+} 0.5 mol% doped $Y_3Al_5O_{12}$ CPP with high blue incident power density. (a) luminous emittance (b) luminous characteristics.
